# Resting-state functional connectivity of the ventral tegmental area and negative symptom domains in subjects with schizophrenia

**DOI:** 10.1192/j.eurpsy.2021.1447

**Published:** 2021-08-13

**Authors:** P. Pezzella, G.M. Giordano, A. Perrottelli, G. Cascino, F. Marciello, G. Blasi, L. Fazio, A. Mucci, S. Galderisi

**Affiliations:** 1 Department Of Psychiatry, Università Campania Luigi Vanvitelli, Naples, Italy; 2 Department Of Medicine Surgery And Dentistry - Section Of Neuroscience, University of Salerno, Salerno, Italy; 3 Department Of Basic Medical Science, Neuroscience And Sense Organs, University of Bari ‘Aldo Moro’, Bari, Italy

**Keywords:** schizophrénia, negative symptoms, functional magnetic resonance, functional connectivity

## Abstract

**Introduction:**

Negative symptoms (NS) represent a core aspect of schizophrenia with a huge impact on real life functioning. Dysfunctions within the dopaminergic cortico-striatal circuits have been documented in subjects with schizophrenia (SCZ) and hypothesized as possible neurobiological mechanisms underlying some domains of NS.

**Objectives:**

We investigated relationships between the resting-state functional connectivity (RS-FC) of the ventro-tegmental area (VTA) and NS.

**Methods:**

Resting-state fMRI data were recorded in 35 SCZ, recruited within the Italian Network for Research on Psychoses. We performed partial correlations between RS-FC and NS (evaluated with the Brief Negative Symptom Scale) controlling for possible sources of secondary negative symptoms.

**Results:**

We found that the experiential domain correlated with the RS-FC of the VTA with the left ventro-lateral prefrontal cortex (lVLPFC) (r=0.372, p=0.039), while the Expressive deficit domain correlated with the RS-FC of the VTA with the left dorso-lateral prefrontal cortex (lDLPFC) (r= 0.470, p .008). Looking at subdomains, only the avolition (r= 0.418, p=0.019) and the blunted affect (r= 0.465, p=.008) showed the same correlations of the domains to which they belong.

**Conclusions:**

According to our findings, separate dysfunctional neuronal circuits could underpin distinct negative symptom subdomains. A better understanding of neurobiological dysfunctions underlying NS could help to design new treatments, targeting different NS subdomains.

